# Successfully managed aluminum phosphide poisoning: A case report

**DOI:** 10.1016/j.amsu.2021.102868

**Published:** 2021-09-17

**Authors:** Srijana Katwal, Kiran Malbul, Sujit Kumar Mandal, Soniya KC, Md Zafar Alam, Parag Karki, Chiranjibi Pant

**Affiliations:** a Shree Birendra Hospital, Chhauni, Kathmandu, Nepal; b Department of Medicine, Resident of Internal Medicine, Shree Birendra Hospital, Chhauni, Kathmandu, Nepal; c Department of Medicine, Faculty of Internal Medicine, Shree Birendra Hospital, Chhauni, Kathmandu, Nepal

**Keywords:** Aluminum phosphide, Case report, Poisoning, Refractory shock, Suicide

## Abstract

**Introduction:**

and Importance: Aluminum phosphide (ALP) is a commonly available pesticide in agricultural countries like Nepal. Upon ingestion, this releases highly toxic phosphine gas in the gastrointestinal tract when it comes in contact with humidity. This leads to refractory shock, metabolic acidosis, cardiac arrhythmia, renal failure, and hepato-biliary impairment.

**Case presentation:**

We present a successfully managed case of a 17-year-old girl who ingested 6 g (2 tablets) of ALP tablets with suicidal intent. Although the mortality has been reported as 70–100% with mere ingestion of 150–500 mg of ALP, this case survived even after developing severe metabolic acidosis, acute renal failure, refractory shock, and ventricular tachycardia.

**Clinical discussion:**

ALP poisoning is most often lethal. However, there is an emerging evidence of successful use of various drugs such as magnesium sulfate, trimetazidine, and other interventions such as intra-aortic balloon pump and extra corporeal membrane oxygenation in case of ALP poisoning.

**Conclusion:**

Owing to the unavailability of an effective antidote of ALP to date, we emphasize early initiation of supportive management, intensive monitoring, and potential role of membrane stabilizers like magnesium sulfate, and cardio-protective agents like trimetazidine, *N*-Acetyl cysteine, thiamine, vitamin C, and hydrocortisone in decreasing the likelihood of fatal outcome.

## Highlights

• Aluminum phosphide poisoning poses a major risk of mortality as it has no specific antidote.
• Early supportive management, critical monitoring, and timely intervention of shock and arrhythmia can save a life.
• Magnesium sulfate, trimetazidine and *N*-Acetyl cysteine may add additional advantages in combating the fatal outcome.

## 1 Introduction

Aluminum Phosphide (ALP) is a highly toxic pesticide that has been widely used in the field of agriculture for crop protection. ALP is a widely and easily available, cheaper chemical in agricultural countries like Nepal due to which the case of ALP poisoning has been increasing in the last few decades [1].

ALP, when ingested, releases phosphine gas which causes non-competitive inhibition of electron transport chain in cytochrome oxidase leading to diffuse cellular hypoxia [2]. The patients often present with sudden onset of abdominal pain, vomiting, refractory hypotension, and arrythmias [2,3]. To date, there is no proven antidote of ALP poisoning and management is symptomatic [3]. The lethal dose of ALP is 150–500mg [2] and mortality is very high which is documented to be about 70–100% [4].

We present a case of successfully managed suicidal consumption of 6 gm of ALP by a 17-year-old girl.This case report is in line with CARE 2017 criteria [5].

## 2 Case Presentation

A 17-year young girl with no known psychiatric illness presented to the emergency department after alleged ingestion of two tablets of ALP with suicidal intent. The patient experienced shortness of breath, multiple episodes of vomiting, and abdominal discomfort shortly after ingestion. She was rushed to a nearby hospital, where she received a gastric lavage with potassium permanganate about half an hour after consuming ALP. She was referred to our center for further management after her hemodynamics were stabilized. The patient arrived at our center 5 h after ingesting ALP. She was conscious but hemodynamically unstable on examination, with barely recordable blood pressure. Her body temperature, pulse, and respiratory rate (RR) were all normal, and she had 91% oxygen saturation in room air.

**Fig. 1:**
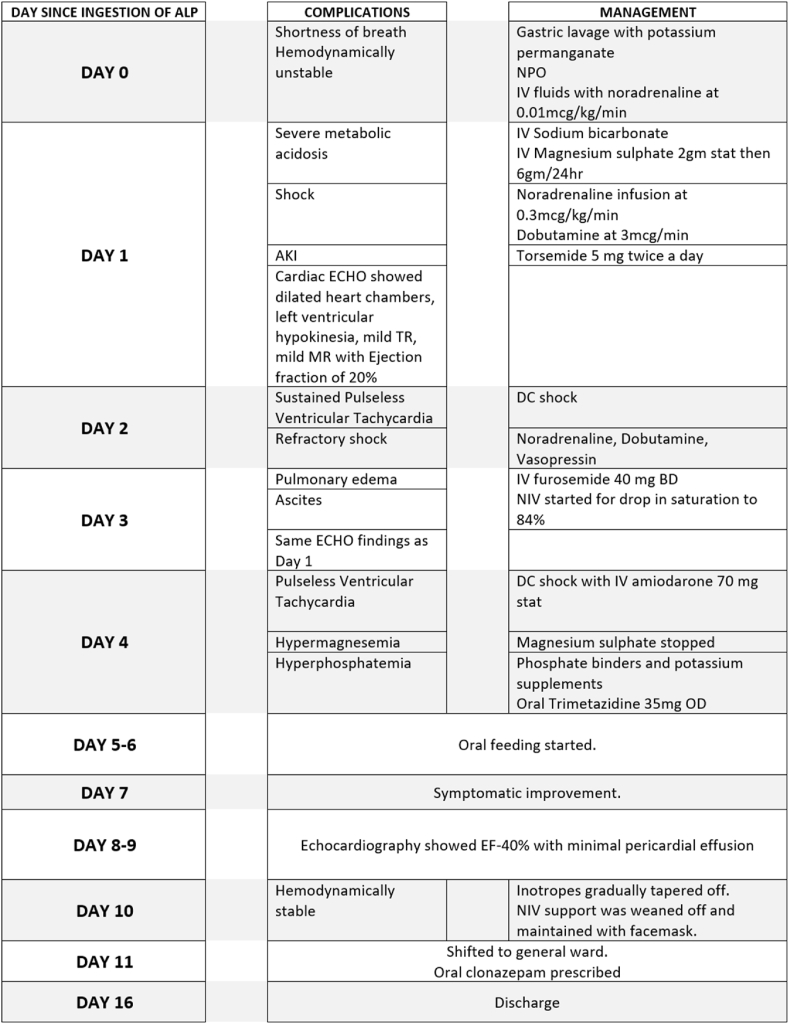
Summary of complications and management since ingestion of 6 gm of aluminum phosphide.

She was kept nil per oral to prevent further release of phosphine gas. Guarded fluid therapy along with noradrenaline at the rate of 0.01 mcg/kg/min was immediately started in an attempt to maintain mean arterial pressure (MAP). She was then moved to the intensive care unit (ICU) for further monitoring and treatment. Fig. 1 depicts a timeline of events following the ingestion of ALP.

On her first day in the ICU, she developed severe metabolic acidosis [pH: 6.855, pCO_2_: 17 mmHg, HCO_3_^−^:3.0mmol/L, lactic acid: 15.63mmol/L]. Despite continuous fluid challenge and noradrenaline infusion titrated at 0.3 mcg/kg/min, her blood pressure was still unrecordable for which dobutamine 5 mcg/min and up titrated to maximum dose was added. Oxygen supplementation and intravenous sodium bicarbonate (NaHCO_3_) were started to manage hypoxia and metabolic acidosis respectively. Intravenous magnesium sulfate (MgSO_4_) 2 gm IV stat and 6 gm over 24 hours was also initiated. Echocardiography showed all heart chambers dilated, global left ventricular hypokinesia, right ventricular systolic dysfunction, mild tricuspid and mitral regurgitation, ejection fraction (EF) of approximately 15–20%, and dilated inferior vena cava of <50% compressibility.

On the second day, she developed refractory shock and was stepped up to triple inotropic support (noradrenaline, dobutamine, and vasopressin). Her liver functions were deranged with alanine transaminase (ALT) 526.5U/L, aspartate transaminase (AST) 913.2U/L, alkaline phosphatase (ALP) 58U/L, total bilirubin 1.35mg/dl, and direct bilirubin 1.09mg/dl. Serial liver function tests showed gradual improvement and no active intervention was required. She also developed acute kidney injury (AKI) with 24-h urinary output decreased to 500ml, plasma urea 74mg/dl, and plasma creatinine 2.71mg/dl for which torsemide 5mg twice daily was prescribed. This was aided with antioxidants like *N*-Acetyl cysteine (NAC), thiamine, vitamin C, and 200mg hydrocortisone in 24-h continuous infusion.

She developed sustained pulseless ventricular tachycardia (VT) on the second and fourth days of treatment, as shown in figure, which was reverted by DC shock and intermittent unsustained VT, which was treated with an intravenous amiodarone 150mg stat dose. Even after 48 hours of ICU stay, the patient showed no signs of improvement and also developed hypermagnesemia [Mg^++^: 4.05mg/dl] and hyperphosphatemia [PO_4_^−-^: 6.35mg/dl]. So, injection MgSO_4_ was discontinued, and cytoprotective agents such as oral trimetazidine (35mg once daily dose); phosphate binders and potassium supplementation for electrolyte balance were introduced. Due to the family's financial constraints, other forms of supportive treatment such as extracorporeal membrane oxygenation (ECMO) were not considered.

On day 3, ultrasonography of the abdomen revealed mild to moderate ascites, mild bilateral pleural effusion, and mild bilateral renal parenchymal echogenicity. Despite using a reservoir mask to provide oxygen, her oxygen saturation dropped to 84–88%, necessitating the use of noninvasive ventilation continuous positive airway pressure (NIV-CPAP). An echocardiogram was repeated on day 3, which revealed the same results as day 1. Thus, a restrictive fluid strategy and diuretics were initiated, to prevent heart failure and fluid overload.

On day 5, oral feeding with liquid sips and a semi-solid diet was resumed. The patient began to improve symptomatically on day 7. On day 9, her echocardiography revealed an EF of about 40%, increased global cardiac contractility, normal chamber size, and minimal pericardial effusion. She was hemodynamically stable, and the inotropes were gradually tapered off and stopped on day 10. NIV support was weaned off successfully and oxygen saturation was maintained with a facemask. She was transferred to the general ward on the 11th day of her admission. After psychiatric consultation, oral clonazepam was prescribed for deliberate self-harm. On day 16, the echocardiographic finding was found to be normal. Then the patient was discharged on oral trimetazidine and aldactone with advice for follow-up in 7 days.

### 2.1 Follow-up

Follow-up was carried out 1-month post hospitalization via teleconsultation which revealed that patient currently is asymptomatic and not under any medication.

## 3 Discussion

ALP, wheat pills/rice pills, is one of the common poisoning seen in South Asia including Nepal [6]. Ingestion and inhalation are the two frequently observed routes of poisoning with suicidal intention more than accidental or occupational exposure.

Upon ingestion of ALP, it reacts with gastric acid in the stomach producing a lethal gas “phosphine” which readily diffuses to various tissue. Phosphine, a protoplasmic poison, disrupts mitochondrial oxidative phosphorylation, inhibits cytochrome C oxidase, and inhibits the production of cell enzymes and proteins leading to cellular hypoxia, decrease ATP production and activation of peroxide radicals [7].

Out of 140 fatal cases of ALP poisoning studied in Albania, 81 were females and 59 were males, with a mean age of 35 years. Female adolescents were found to be at a higher risk of poisoning in the same study [8]. Similarly, an adolescent girl ingested ALP tablets with a suicidal motive in our case.

ALP poisoning may have variable manifestations ranging from nonspecific symptoms and signs like vomiting, abdominal pain, loose motion, severe thirst, tachycardia, tachypnea and shortness of breath to the life-threatening severe metabolic acidosis, shock, arrhythmias, acute renal failure, disseminated intravascular coagulation, and acute respiratory distress syndrome causing death within 24 hours in most of the cases [9]. Cardiovascular complications like reduced ejection fraction, refractory hypotension, and electrocardiographic (ECG) abnormalities such as arrhythmia, ST-T wave changes, and conduction defects are seen in ALP poisoning [9]. Few studies have mentioned sound mental status until the patient develops cerebral anoxia following shock which may later lead to drowsiness, delirium, and coma [7,10]. Refractory shock is seen in 60%–100% of cases which is attributed to the direct effect of phosphine on cardiac myocytes, adrenal gland, and blood vessels resulting in peripheral vasodilation, fluid loss, and dehydration [11,12]. Similar findings were observed in our case where the patient presented in a conscious state, experiencing shortness of breath, vomiting, abdominal discomfort, and severe thirst with barely recordable blood pressure. And later on, she developed refractory shock, severe metabolic acidosis, and acute kidney injury.

As there is no specific antidote for ALP poisoning, all cases should be treated according to general poisoning management principles. Early resuscitation, decontamination, gastric lavage with potassium permanganate/coconut oil, preferably within 1–2 hours, oxygenation, and other symptomatic management are all part of this. MgSO_4_ stabilizes the cell membrane and helps to combat the effects of released free radicals [13]. Daily monitoring of magnesium is recommended to prevent hypermagnesemia [13]. NAC has been shown to reduce hospitalization time, intubation, and mortality [14]. Trimetazidine, a cardio-protective anti-ischemic drug, preserves oxidative metabolism. This drug decreases intracellular calcium and increase intracellular ATP via various mechanism and prevents myocardial injury [9]. Duenas et al. has highlighted the successful use of trimetazidine in the ALP poisoned case [15]. Here, in this case, we prescribed MgSO_4_, NAC, vitamin C, and trimetazidine.

She had two episodes of sustained VT, both of which reverted. In a study by Siwach et al. among all ALP poisoned cases, about 40% had ventricular tachycardia and 23.3% had ventricular fibrillation [16]. Echocardiographic findings in ALP poisoned cases revealed left ventricle wall motion abnormalities, decreased EF, and pericardial effusion [7] as in our case.

Phosphine gas is known to cause damage to the renal and hepato-biliary systems as well [13]. Our patient developed AKI within the first 48 hours of admission, which improved later on. Elevated AST and ALT are the most common signs of hepatocellular impairment, and the same is seen in our case. Jaundice is another sign of liver disease, but it is uncommon [13].

Vitamin C and E, melatonin, glutathione, beta-carotene, and dihydroxyacetone have also been proposed for the treatment of ALP poisoning, but their efficacy requires more research [17]. Other treatments for persistent refractory shock in ALP poisoning include induction of hyperinsulinemia-euglycemia [18]; intra-aortic balloon pump [19], pacemaker implantation, and ECMO [20].

## 4 Conclusion

Although ALP poisoning has a high mortality rate, we were able to successfully manage the case, which could be attributed to early intensive and supportive management with MgSO_4_, trimetazidine, NAC, thiamine, vitamin C, and hydrocortisone. This case adds additional evidence of successful management of ALP poisoning.

## Sources of funding

This study did not receive any kinds of grant or fund.

## Ethical approval

N/A.

## Consent

Written informed consent was obtained from the patient for publication of this case report and accompanying images. A copy of the written consent is available for review by the Editor-in-Chief of this journal on request.

## Author contribution

All authors approved the final version of the manuscript and agreed to be accountable for all aspects of the work in ensuring that questions related to the accuracy or integrity of any part of the work are appropriately investigated and resolved.

## Registration of research studies

1. Name of the registry: N/A.

2. Unique Identifying number or registration ID: N/A.

3. Hyperlink to your specific registration (must be publicly accessible and will be checked): N/A.

## Guarantor

Srijana Katwal, Shree Birendra Hospital, Kathmandu, Nepal, Email: drsrikknepal@gmail.com.

## Patient perspective

Our patient's insight and thought regarding life and suicidal intent have been changed. She is seeking ongoing psychiatric care. At discharge she was appreciative of the entire medical team for her successful recovery.

## Provenance and peer review

Not commissioned, externally peer-reviewed.

## Declaration of competing interest

None declared.
